# Supplementary material for: Asynchronous magnetic resonance elastography: Shear wave speed reconstruction using noise correlation of incoherent waves
Source: Magn Reson Med. 2022 Oct 27;89(3):990–1001. doi: 10.1002/mrm.29502 (PMC9792433; doi:10.1002/mrm.29502)
Supplement: Supplementary file 1 — DATA S1 MATLAB code used to generate the numerical simulations of Figure 1 [file MRM-89-990-s002.zip › k-Wave/helpfiles/waterNonlinearity.html]

waterNonlinearity :: Functions (k-Wave)


# waterNonlinearity

Calculate B/A of water with temperature.

## Syntax

```
BonA = waterNonlinearity(T)
```

## Description

`waterNonlinearity` calculates the parameter of nonlinearity B/A at a given temperature using a fourth-order polynomial fitted to the data given by Beyer (1960, Parameter of nonlinearity in fluids, J. Acoust. Soc. Am., 32(6), 719-721). A plot of the original data and the polynomial fit is given below.

## Inputs

|  |  |
| --- | --- |
| `T` | water temperature in the range 0 to 100 [degC] |

## Outputs

|  |  |
| --- | --- |
| `BonA` | parameter of nonlinearity |

## See Also

`waterAbsorption`, `waterDensity`, `waterSoundSpeed`
